# Skill building in freediving as an example of embodied culture

**DOI:** 10.1098/rstb.2023.0150

**Published:** 2024-10-07

**Authors:** Greg Downey

**Affiliations:** ^1^ School of Social Science, Macquarie University, Sydney, New South Wales, Australia

**Keywords:** apnoea, embodiment, canalization, freediving, biocultural anthropology

## Abstract

Skilled activity is a complex mix of automatized action, changed attention patterns, cognitive strategies and physiological adaptations developed within a community of practice. Drawing on physiological and ethnographic research on freediving, this article argues that skill acquisition demonstrates the variety of mechanisms that link biological and cultural processes to produce culturally shaped forms of embodiment. In particular, apneists alter phenotypic expression through patterned practices that canalize development, exaggerating the dive response, developing resistance to elevated carbon dioxide levels (hypercapnia) and accommodating hydrostatic pressure at depth. The community of divers provides technical advice and helps to orient individuals’ motivations. Some biological processes are phenomenologically accessible, but others are sub-aware and must be accessed indirectly through behaviour or altered interactions with the environment. The close analysis of embodied skills like freediving illustrates how phenotypic plasticity is inflected by culturally patterned behaviours. Divers do developmental work on bodily traits like the dive response to achieve more dramatic performance, even if they cannot directly control all elements of the neurological and physiological responses. The example of expert freediving illustrates the imbrication of biology and culture in embodiment.

This article is part of the theme issue ‘Minds in movement: embodied cognition in the age of artificial intelligence’.

## 1. Introduction

Cross-cultural variation in bodily practices has long attracted the attention of anthropologists. Spectacular forms of cultural elaboration, like body alteration, arduous rituals and elaborate adornment, have underscored that the body is a cultural object not just a biological fact [1,2]. However, compelling evidence of embodied variation also comes from extremes of human achievement in sports and expert performance. Athletes and other highly skilled experts demonstrate how our species’ adaptability can be stretched not only by environmental or selective pressures but also by diligent training regimens. Expert individuals devote themselves in communities of practice to regimes of intentional bodily cultivation over extended periods with dramatic neurological, biomechanical and anatomical consequences.

Skilled activity in sport is a complex mix of automated action, refined attention patterns, sophisticated cognitive strategies and physiological adaptation, learnt from and developed within social groups (just as complex behaviours develop in all species albeit in more elaborate form). For this reason, athletic populations are an ideal field to realize the holistic ambition of anthropology to unite biological and cultural approaches to human variation [3, p. xiv; 4–6].

The division of anthropology into biological and cultural subfields, however, has awkwardly bifurcated the discussion of embodiment in our discipline, including the analysis of skill acquisition, leading some researchers to treat biological and cultural explanations as competing rather than necessary complements [7]. At its best, anthropology can provide synthetic, holistic accounts of human development and variation, drawing on a wealth of cross-cultural case studies, sociocultural theory and unmatched comparative biological data amassed by our field over more than a century [8–11]. However, a persistent version of the ‘two-cultures’ war [12] flares up sporadically in the field as some anthropologists seek to maintain a ‘no man’s land’ between biological and cultural accounts, making synthesis more difficult.

The bifurcation often leads cultural researchers to hesitate at the border separating behavioural from biological research, even though cultural practices have clear physiological and neurological consequences. As Lock warns, ‘the interiority of the material body has been normatively “black-boxed” by (socio-cultural) anthropologists on the assumption that this is the domain of biologists and physical anthropologists and beyond the purview of social scientists’ [13, p. 457; 14]. In this context, cultural anthropologists labelling change ‘embodiment’, ironically, can be a way to avoid engaging with the biological consequences of culture rather than an opportunity for robust synthetic accounts of the imbrication of physiology and culturally conditioned experience.

Although skills like walking, running and swimming arise reliably in human development, these techniques take different forms cross-culturally, as Mauss observed [15]. Ingold argues that this behavioural variation has physiological consequences because ‘throughout life’, the human body ‘undergoes processes of growth and decay, and…as it does so, particular skills, habits, capacities and strengths, as well as debilities and weaknesses, are enfolded into its very constitution—in its neurology, musculature, even its anatomy’ ([16, p. 26]; refer also to [9,17,18]). Classic examples from biological anthropology include the effect of shoes on the anatomy of the foot [19,20], upper limb asymmetries from uneven loading [21,22] and distinctive ankle facets from habitually resting in a squat [23,24].

Roepstorff *et al.* advocate defining culture itself as ‘patterned practice’ [25]. This definition does not capture all aspects of human lifeways, but it does suit especially well studying the embodied consequences of diverse training regimes [26]. Recognizing that cultural practice is a primary driver enculturing human embodiment leads Lende and Downey ([27,28]; refer also to [29]) to characterize reciprocal causation at the biocultural interface as a ‘behavioural-developmental spiral’: patterned behaviour canalizes developmental plasticity while phenotypic refinement underwrites skilful, encultured activity. This article seeks to show that the close analysis of the biocultural interfaces in freediving demonstrates the mechanisms engaged by patterned practice in training and the complex interaction of social, psychological, perceptual, neurological and physiological factors intertwined in the patient cultivation of skill.

## 2. The emergence of freediving as a community of practice

Freediving has been practised widely since antiquity, with traditions of pearl divers, ocean foragers, underwater salvage and military diving in the Mediterranean, East Asia, Australia and the Pacific. The sport of freediving or competitive apnoea, however, with its distinctive array of events emerged in Europe in the twentieth century. Umbrella organizations dedicated to underwater sports were established, most prominently in 1959, the Confédération Mondiale des Activités Subaquatiques (CMAS, World Underwater Federation). Conflict over appropriate regulation within the umbrella organizations, especially of high-risk events, led to the founding of specialized groups like the Association Internationale pour le Développement de l’Apnée (AIDA, International Association for the Development of Apnea) in 1992. A global circuit of international competitions now exists with multiple promoters and organizers for apnoea training, competitions and regulation.

The establishment of regulated events, with carefully designed safety measures to protect divers, together with sophisticated training techniques and advances in scientific understandings of hypoxia, led to rapid increases in world records in freediving over the last 70 years. In the early twentieth century, medical experts warned that if the lungs were compressed below their ‘residual limit’, the minimum size a person can achieve by exhaling—reached around 50 m depth—the lungs might bleed or irreversibly collapse [30, p. 587]. By 1968, however, physiologists like Craig [30] pointed out that divers were descending below this theoretical limit [31,32]. Robert Croft, for example, dove to 80 m in 1968, and Jacques Mayol descended below 100 m in 1976. These athletic pioneers simultaneously contributed to scientific studies of human adaptation to diving [33]. The traffic is two ways: practitioners actively adapt scientific insights into practice and borrow from other forms of training. Mayol, for example, helped to introduce stretching and breathing techniques from yoga to freediving [33, pp. 9–10].

As divers reach greater depths, pressure on the body builds: every 10 m of depth adds one atmosphere of hydrostatic pressure. This pressure can damage the ears, sinuses, lungs, airways and eyes, and it complicates dive safety because rescue divers also have to contend with depth. At the same time, as divers go deeper than 15 or 20 m, they become negatively buoyant, essentially free falling, which aids them to conserve oxygen by permitting even greater relaxation. Divers who go to depth can lose consciousness unexpectedly as they ascend: the rapid decrease in pressure causes blood oxygen levels to fall quickly. In many freediving competitions, participants can lose consciousness or suffer other transient symptoms of hypoxia, like loss of motor control or what divers call a ‘samba’. For these reasons, trained rescue divers and medical personnel are necessary, although most incidents do not lead to any injury or long-term effect [34]. Competitions, even though they encourage the deepest dives, are relatively safe, with only two fatalities in many decades of regulated competition (although deaths in training have occurred [35]).

The freediving community is an example of what Lave and Wenger call a ‘community of practice’ [36,37]. Lave and Wenger [36,37] describe how experts and novices form social communities around shared learning projects; Downey specifically applies their approach to the freediving community [38]. Informal educational communities around activities like freediving offer a repertoire of resources for skill-building and bodily modification. Even though freediving is an individual sport, the practice is informed, scaffolded and buttressed by social interaction; the community, through sustained practice, scientific inquiry and borrowing from other sports communities, has built up a distinctive applied understanding of key bodily mechanisms [38]. Moreover, practice is shaped by the activities that are especially highly valued or considered developmentally central. In my experience diving in Australia and Tonga, participants train to freedive for many reasons: photography, sightseeing, exploration, recovering objects on the seabed, spearfishing and surfing more safely. However, diving for depth, usually along a dive line as in competitions, and static breath holds for maximum time were the primary training activities.

Formal dive events are divided into three categories: static, in which divers maintain a breath hold as long as possible while stationary; dynamic, swimming as far as possible horizontally and depth, competing to achieve the greatest depths. The dynamic and depth events are further divided by rules governing the equipment divers can use, such as whether they wear fins or use ballast to aid descent. Most recreational freedivers, however, do not participate in formal competitions, even though competitive freediving is disproportionately influential in research, dive instruction and public awareness of freediving. For example, the most spectacular depth records have been achieved in ‘no limits’ dives, the rarest event, no longer included in freediving competitions. In ‘no limits’ dives, participants use any equipment they choose to speed descent, such as, in the most extreme cases, heavy ballast, or to aid ascent, such as inflatable buoyancy devices. These dives are dangerous, as they often use innovative technology, such as inflatable vests or sleds that drag participants to a pre-determined depth. By 1970, the CMAS largely stopped validating ‘no limits’ record attempts owing to the danger, and the AIDA suspended ratification in 2012 after several participants died. Owing to the extreme pressures at depth, divers risk blackout, lung or trachea damage from ‘the squeeze’ or nitrogen narcosis when returning to the surface. The current depth record for a sled-assisted ‘no limits’ dive is 214 m, held by Austrian Herbert Nitsch; Nitsch was badly injured on a subsequent attempt to break this record, suffering a stroke that left him partially paralysed and in a coma for 7 days Any divers still engaging in ‘no limits’ dives are, in effect, a renegade segment of extreme practitioners within a larger community of athletes, which is itself a subset of a broader community of freedivers, most of whom do not compete. The point is that the cultural configuration of diving practice in sport apnoea, especially the competitive focus on achieving the greatest depths, as well as the history of injuries in events, affects how divers train and interact with the water and their resulting adaptations, just as this history also influences how scientific knowledge about the human body emerged from collaboration between scientists and athletes. The ‘niche’ to which freedivers’ bodies adapt is defined not only solely by water but also by their culturally patterned behaviours; the developmental niche is an eco-cultural construction [39,40].

The diving niche created by international sport diving diverges radically from other communities of freediving practice. For example, the Bajau-Laut people of Southeast Asia often dive to forage on the sea floor and fish underwater; they perform frequent, short dives, with minimal recovery time over many hours (like spear fishers in the Australian context). In addition, most members of some Bajau-Laut communities dive, a practice that has persisted across multiple generations. This cultural pattern of diving produces a different suite of phenotypic adaptations, including apparent genetic selection for enlarged spleens [41,42]. Another group of sea foragers, the Moken, who search the ocean floor starting in childhood, develop non-conscious visual behaviour for more acute underwater vision, an adaptation that would not arise among sport freedivers who sometimes with their eyes closed in-depth events [43]. The ‘sea women’, the Ama divers of Japan and Haenyeo of Korea, by contrast, endured severe cold, diving unprotected in water temperatures as low as 13 to 14°C before the introduction of neoprene wetsuits in the 1970s [44,45]. These women demonstrated circulatory and metabolic adaptations, including particularly dramatic vasoconstriction to sequester heat in their bodies’ core [46]. Some of the adaptations overlap with sport freedivers, but others are distinctive because of the configuration of diving behaviours in diverse contexts.

The author’s direct experience of freediving and physiological changes is limited to practising as a hobbyist for 2 years, starting in 2017. Most of the research supporting this article was secondary research, conducted from 2020 to 2022, affected heavily by pandemic restrictions in Australia. For this reason, the article is an ethnographically informed theoretical synthesis drawing on a broad reading of specialist freediving materials (manuals, websites, divers’ accounts and practitioners’ discussion boards) and diverse scientific literature (from physiology, medicine and human biology). The challenges of synthesizing this breadth of material are familiar to anthropologists.

## 3. Reinforcing the dive response with patterned practice

During a breath hold in water, the human body typically has a dive response, a cluster of reactions found across vertebrates that protects the body, especially the brain, when respiration ceases [47,48]. When humans dive, the body undergoes three major changes. First, the nervous system initiates bradycardia: the heart rate slows, especially when cold water covers the sinuses. Second, the peripheral arteries narrow (vasoconstriction) and shunt blood to the brain and vital organs [49]. The combination of the two maintains adequate blood pressure as the heart rate drops. Third, the spleen contracts, expelling extra oxygen-carrying cells into the bloodstream [50]. Infants demonstrate the dive response when immersed, although research suggests that the response becomes less pronounced beyond six months of age if not reinforced [51].

The dive response is homologous across mammals with similar responses in fishes and birds and is mediated by areas in the medulla and the spinal cord, so, like tidal breathing, it is highly automated. Diving mammals, such as seals and sea lions, possess additional anatomical and neurological adaptations for diving, such as elevated blood volumes, enlarged spleens, collapsible lungs and better control over blood flow; these animals undergo dramatic metabolic changes when diving [52]. Although humans do not have the same specialized adaptations, the human dive response can be trained to a remarkable degree, making apnoeas possible that go well beyond normal physiological limits (the current record for static apnoea without breathing pure oxygen in preparation is 11 min 35 s [53–55]). The degree of bradycardia in a human dive response varies with age and training, from virtually no decrease to dramatic reductions [56,57]. Schagatay suggests that untrained subjects typically decrease their heart rate 30 to 40% during an apnoea with facial immersion [48, p. 135]. Gooden found that 10 to 15% of healthy, young test subjects were ‘marked responders’, demonstrating greater bradycardia [58]. By contrast, some inexperienced subjects demonstrate a maladaptive ‘defensive’ tachycardia response when submerged; their heart rates accelerate, typical of a stress response and opposed to the protective dive response [59]. The maladaptive defensive response can be extinguished through habituation.

Elite divers’ heart rates can drop as low as 20–30 beats per minute or around half normal, including some of the lowest heart rates on record; in addition, bradycardia triggers more quickly in experts [54,60]. Research with freedivers found they have stronger vagal tone, the nervous activity that drives the parasympathetic system, linked to slowed heart and emotional regulation [61]. Schagatay points out that these decreases (around 50% for experts) are similar to dive bradycardia in beavers, muskrats and manatees, aquatic or semi-aquatic mammals but not deep divers [48, p. 135].

To prime bradycardia, divers typically perform preparatory activities: some meditate, dunk their faces repeatedly to trigger the sensors in the face and perform relaxation exercises. These preparations make use of phenomenologically apparent proxies linking emotional states and volitional control of breathing through sensory receptors to the sub-aware parasympathetic system. Divers avoid any excitement that might increase heart rate and deplete oxygen in the bloodstream [58].

Alongside bradycardia, divers also undergo sympathetically induced vasoconstriction in the peripheral circulatory system [49]. Vasoconstriction reduces blood flow to the extremities, shunting blood to the body’s core and maintaining blood pressure as heart rate drops. In animals adapted for diving, vasoconstriction is severe: anticipating a long dive seals dramatically decrease blood flow to muscles and visceral organs, only circulating oxygenated blood to the brain [52, p. 10]. As with bradycardia, vasoconstriction varies among human subjects; research found more than five times greater vasoconstriction in experienced divers compared to controls, so it may be more susceptible to training effects than bradycardia, even though it is less phenomenologically salient to divers [62, p. 723; 63]. The modification in circulation is so profound that physiologist Gooden discovered blood flow almost stopped to the forearm of a subject who underwent a particularly dramatic dive response [58, p. 7].

The example of novice divers with maladaptive responses underscores the fact that veteran freedivers have more efficient, better-articulated dive responses than novices [57]. In the most extreme cases, ‘autonomic conflict’ may arise between parasympathetic bradycardia and sympathetic tachycardia, opposed cardiac responses. Shattock & Tipton [64] draw on evidence of arrhythmias in healthy subjects immersed in cold water to offer a potential explanation for the phenomenon of ‘dry drowning’: drowning victims found to have no water in their lungs when autopsied. They argue that strong simultaneous activation of sympathetic and parasympathetic responses in sudden cold-water immersion may overwhelm the heart. Even in expert divers, dyssynchronous heart rhythms, possibly from autonomic conflict, can create heart arrhythmias, although these patterns are also seen in some deep-diving mammals, suggesting the response is neither wholly adaptive nor maladaptive [54, pp. 1348–1349]. The point is that the dive response, under training conditions, can be reinforced, but it also can be undermined, including by individuals’ emotional experiences or behaviour.

An expert dive response is a combined biological and cultural achievement. Built upon ancient neurological potential, reflex patterns that predate our species’ origins, it also involves volitional self-regulation of emotions, bodily priming through patterned behaviour, community practical and scientific learning, and even escalating performance records and competition formats with failsafe procedures that mitigate divers’ risks of engaging in dangerous behaviour. Expert divers and the community of practice study their bodies’ reactions and their performances reflexively. They seek to influence their circulatory systems even though they have incomplete volitional control or even perception, recognizing factors that trigger or disrupt bradycardia and vasoconstriction, including mental and emotional activity [65,66]. In the case of the dive response as embodied culture, training techniques and patterned practice build upon a deeply conserved neurological capacity, reinforcing reactions that are not entirely under participants’ control.

## 4. Interoceptive learning and affective executive control

The subjective experience of diving is crucial: bradycardia is affected by emotional states, and divers must also confront both the urge to breathe and the diaphragm’s attempts to restart respiration as they push beyond their habitual interoceptive limits to apnoea duration. As carbon dioxide accumulates in the bloodstream, the diaphragm muscles eventually clench to try to restart breathing in what divers call a ‘contraction’ [67]. Contractions recur with increasing frequency, sometimes only seconds apart; the subjective experience of contractions varies, with some divers suffering significant, increasing discomfort as the diaphragm becomes more active. Researchers have referred to these activations of inspiratory muscles triggered by hypercapnia (elevated CO_2_) as ‘involuntary ventilatory activity’ (IVA) [68] or ‘involuntary breathing movements’ [69]. Dejours called the first contraction the ‘gasping point’, which marked the transition from the ‘easy phase’ of an apnoea to the ‘struggle phase’ [70, p. 183]. Schagatay refers to this second part of a breath hold as the ‘fighting phase’, in which the ‘urge to breathe imposed by the combined stimuli of hypercapnia and hypoxia requires a strong effort not to resume breathing’ [71, p. 92].

Lin *et al.* argued that the onset of IVA was the longest a ‘naïve diver’ would voluntarily endure a breath hold [68]. The implication is that sustaining apnoea through IVA into the ‘struggle phase’, during which the diver experiences repeated contractions, required coaching or advice from a more experienced diver. Freediving training includes relaxation and distraction techniques to help divers endure multiple contractions, and some divers report the subjective distress decreases significantly with training. The contraction sensation was specifically discussed and pedagogically embedded in apprenticeship; coaches reassured inexperienced divers that contractions would grow less severe with training, and discussions of contractions occur in every diving manual the author reviewed. During one in-pool session, the author attended, experiencing a contraction—ideally, continuing an apnoea through multiple contractions—was an explicit goal for an exercise. Diving manuals offer advice to inexperienced divers about how to think about contractions to diminish their effect (e.g. [72]). The embodied mechanism that permits a diver to overcome a contraction is an inhibitory executive act of self-control against the urge to breathe, informed by culturally available information, including scientific data sometimes, which reassures the novice that disregarding the sensation will not cause self-harm.

Although the struggle phase can be painful for some novices as the intensity of IVA increases, in expert apneists, discomfort is delayed, reduced or even extinguished. Accomplished freedivers can withstand more than a hundred contractions before terminating an apnoea [60]. The blood gas level at which trained divers experience the onset of IVA varies, as does the time of onset [57]. For an expert diver, severe hypoxia and impending lung collapse (atelectasis) from hydrostatic pressure—not any discomfort caused by hypercapnia—typically determine the limit to dives or breath-hold duration [73]. Some divers approach 50% blood oxygen levels, the theoretical minimum before blackout, and blackouts do occur regularly in competitions; medical researchers once considered it impossible to hold one’s breath until unconsciousness. Repeated exposure to hypercapnia has been found to blunt ventilatory responses in a range of divers [69], including underwater hockey players [74], submarine escape tower instructors [75] and Ama divers in Japan [76].

Even short-term training, such as a single session of repeated apnoeas, can lead to longer breath holds before hypercapnia can be blunted [77,78]. Novice breath holders who underwent dry static apnoea training evidenced significant gains in breath-hold times after only two weeks of training, even though the onset of the struggle phase was not delayed [79]. According to Bourdas & Geladas, improved performance following short training programmes is attributable primarily to volitional factors, that is, the novices’ increased ‘stamina’ or ‘tolerance’ for the discomfort of the struggle phase [79].

Veteran divers claim to experience decreased struggle as apnoea lengthens, whether because IVA diminishes or hypoxic intoxication occurs. According to Binks *et al.*, the experts they interviewed all used cognitive self-checks during extended apnoeas to avoid losing consciousness, especially after 6–9 min [80]. Divers reported doing mental arithmetic and checking if colour vision still functioned, for instance, to alert themselves to impending blackout, which would disqualify them in competition and necessitate rescue.

To tolerate contractions, blunt the ventilatory reflex and desensitize oneself to higher-than-normal blood acidification, in more general terms, involves a mechanism of ‘perceptual learning’, a change to sensory acuity over time [81]. The apneists’ perceptual learning, however, is peculiar; most perceptual learning involves patterns of motivated attention refining acute exteroception, like heightened visual acuity or hearing sensitivity to task-specific cues. Freediving, however, requires *decreased* interoceptive chemosensitivity rather than finer-tuned discrimination. For most people, chemosensitivity only manifests as discomfort with elevated ventilatory demand, like during vigorous exercise, standing up suddenly or travelling to high altitudes. Elevated tolerance of hypercapnia in apneists is an embodied effect of culture as patterned practice, the biasing of neurological and psychological sensitivity by systematic exposure with empirically observable consequences for automatized bodily reactions.

Proprioceptive change in dive training is also necessitated by the threat hydrostatic pressure poses to the ears. As divers descend, they must equalize inner ear pressure with the surrounding water. Dives as shallow as 4 or 5 m can damage the eardrum if divers do not equalize successfully, and diving vertically in a head-down position along a dive line, as divers do in the depth disciplines in competitions, can make equalization more difficult. Novices often struggle for a variety of reasons; if we felt pain in our ears in the classes I attended, we were told to stop along the dive line until we equalized or return to the surface if unsuccessful. Some coaches advise equalizing every metre as a diver descends and pressure increases rapidly, so these forms of bodily self-manipulation are essential. Some equalization methods require holding the nose, like the Valsalva, Toynbee and Frenzel techniques; ‘voluntary tubal opening’ is a hands-free technique involving pushing air held in the mouth through the Eustachian tubes by shifting the jaw. Whichever technique they use, freedivers must equalize quickly and repeatedly, and some techniques are said to waste air, so equalization can severely limit divers’ ability to reach depths. Middle ear barotrauma is common in professional divers (including scuba divers), with self-reported surveys finding incidence over 80% [82].

The examples of equalization techniques and divers developing the ability to endure diaphragmatic contractions illustrate the importance of internal perceptual dynamics and self-control as forms of embodied change in diver training. Socio-cultural factors, including practical and scientific knowledge shared through the community of practice, enable divers to reinterpret interoceptive sensations and support them gaining greater control over subtle bodily action, such as opening the Eustachian tubes. These changes cannot be learnt simply by imitation; they are invisible, especially given the context in which they are used, underwater and wearing diving equipment. In this setting, instruction must be explicit, even delivered in classroom settings prior to entering the water. In these cases, embodied cultural change is perceptual and behavioural, but it becomes largely automatized and sets the stage for even more arduous change: phenotypic adaptations to gross anatomy.

## 5. Niche-induced phenotypic changes to gross anatomy

As divers maintain apnoeas longer and dive deeper, they confront bodily limitations and, in some cases, extend these limits. In freediving, like in other arduous physical activities, phenotypic development is canalized by behaviour patterns, but divers warn that the body needs time to adapt, for example, to get accustomed to pressure at depth. Enthusiastic novices are cautioned to be patient or risk injury that sets back skill development or even makes continued practice impossible.

Phenotypic development is canalized both by direct mechanical activities—exercises, swimming and stretching—and by indirect environmental influences, as more proficient diving exposes participants’ bodies to increasingly severe environmental stressors. In the freediving community, coaches and veteran divers encourage aspiring novices to perform conditioning exercises outside of dive sessions to improve their performance; these reflexive bodily techniques are especially focused on increasing flexibility in the torso and enlarging lung capacity, but underwater apnoea is the most important conditioning activity [83].

For example, as part of the dive response, shortly after apnoea begins, the diver’s spleen actively contracts [84]. The contraction expels stored red blood cells, boosting oxygen-carrying capacity [48, p. 130; 56, p. 5; 85]. This anatomical response to diving was first observed by Hurford *et al.* in research on divers in South Korea [50]. In animals particularly well adapted for endurance or diving, like horses or seals, the spleen can hold up to half the animal’s red blood cells, reducing cardiac workload when the animal is resting by withdrawing extra cells and lowering blood viscosity [86, p. 361]. Spleen contraction contributes to humans’ response to exercise, apnoea and high-altitude hypoxia [87,88]. Researchers on breath-hold divers have found higher splenic volume in expert divers than non-divers and that spleen size significantly predicts maximum apnoea duration [55,56,88].

Although the cardiovascular system returns to normal quickly after a diver surfaces and breathes, extra blood from the spleen continues circulating, especially if breath holds are repeated. The spleen only starts to uptake haematocrit and haemoglobin about 3 min after a diver’s last apnoea; the surplus is not reabsorbed entirely until at least 10 min rest and possibly longer [89]. Experienced divers are aware of this effect, even if they do not understand the physiology of splenic contraction; they commonly perform ‘warm up’ apnoeas to take advantage of the effect before deep dive attempts.

Whether greater-than-predicted volumes of expert divers’ spleens result from a training effect or from selection for large spleens by sport freediving is not clear. In some experiments, diving appears not to affect spleen size, which some researchers argue is determined more by genetic variation [41,90]. At the same time, some controlled longitudinal research, such as an eight week training programme conducted by Bouten *et al.* [91], demonstrate training effects on spleen volume even after training programmes much shorter than freedivers undergo in the community of practice. Changes to the anatomy of the spleen might require repeated exposure to extreme hypoxia that novices and experimental subjects, because they cannot endure long breath holds, simply cannot achieve.

Lung capacity is affected by training, as researchers found as early as the 1960s with submarine rescue divers and their trainers [92]. Other breath-hold divers evidence lung capacity elevated above what would be predicted given their heights and weights [93,94]. Schagatay, for instance, found that freedivers had on average vital lung capacity 1.8 l greater than control subjects matched for age and body size (an average adult male has about 6 l of lung capacity) [48].

Freedivers deploy a range of reflexive bodily techniques to increase lung capacity, including deep breathing exercises and torso stretches borrowed from yoga. Divers especially use glossopharyngeal insufflation and exsufflation or ‘lung packing’ and ‘reverse packing’ to increase lung capacity. Divers insufflate by gulping air after inhaling as much as possible, ‘packing’ air on top of a full breath. The technique increases lung capacity by as much as 50% in some studies, with most divers achieving 15 to 25% increases [93–95]. Insufflation simultaneously compresses air in the lungs and stretches the tissue, expanding the thorax and displacing the diaphragm downward. The over-inflation leads some medical researchers to warn the technique may cause long-term lung injury, such as pneumomediastinum or air left between the lungs in the chest cavity that can lead to stroke-like phenomena [96–98]. Lung packing, however, may offer some protection against hyperbaria by delaying lung compression below residual volume. It also provides divers with more air to equalize their ears. Diving manuals are ambivalent about insufflation, often describing the practice in detail while also cautioning about potential dangers, but observers find that most divers in competitions insufflate.

Human exhalation relies on passive contraction; to exhale, we simply relax the intercostal muscles that lift and open the ribcage, and the elastic recoil of the torso squeezes the lungs. Seccombe *et al.*, following research on lung capacity and insufflation, suggested that overdistension from repeated bouts of insufflation might temporarily suppress the recoil from the lungs and chest wall [95]. In addition, training in competitive apnoea can increase the strength and control of the intercostal muscles allowing divers to actively distend further when inhaling; even relatively short-term training (11 weeks) was found to increase lung capacity by 0.45 l [48].

Stretching the torso also prepares divers’ bodies for the ardours of depth. As divers descend, the lungs are compressed, although ‘lung packing’ and blood shift can delay the effect. At greater depths, divers must have sufficient flexibility in the diaphragm and ribcage to allow the lungs to be compressed without injury; the lungs of some deep-diving mammals are adapted to collapse completely for protection. Research on divers who dove 56–76 m found 88% experienced haemoptysis afterwards; that is, they coughed blood from pulmonary barotrauma, caused by collapse of alveolar capillaries in the lungs [98,99]. Exsufflation exercises or ‘reverse lung packing’, in which divers exhale as much as possible and then try to squeeze out still more breath, is one technique divers use to improve torso flexibility and guard against barotrauma from ‘the squeeze’. The physiological effects of hydrostatic pressure demonstrate that the difference between ‘adaptation’ and ‘damage’ can be a matter of cultural expectations, including participants’ willingness to take risks and suffer physiological consequences of performance.

## 6. Conclusion

Cultural effects on embodiment are not clearly distinct from biological facts, in spite of what our academic division of labour suggests. As Krieger argues, embodiment is literal [100]. The recognition of cultural variation in embodiment should prompt cultural anthropologists and others to address the complex, multi-directional mechanisms that link social facts, behavioural patterns and ideational constructs in diverse cultures with human phenotypic development. Ingold has observed, however, that even those anthropologists who have accepted the ‘idea of embodiment as a paradigm for the study of culture’ and denounced mind/body dualism often baulk at attempts to integrate biology into cultural research [17, p. 170]. Instead, ‘embodiment’ in cultural anthropology tends to refer to the phenomenological fact of having a body rather than the physiological consequences of patterned practice (e.g. [101]).

One reason is that some anthropologists probably fear, once the ‘black box’ of physiology is opened, it will be difficult to preserve anything ‘cultural’ in a biological account. What the close analysis of mechanisms of embodiment in freediving suggests, by contrast, is that examining the organic consequences of skill acquisition demonstrates instead that enculturation goes deep into the body. Patterned practices affect the nervous system, muscles, connective tissue and functioning of our organism. The mechanisms through which physiology enables cultural expression and through which patterns of practice canalize phenotypic plasticity turn out to be widely varied, multi-directional, operating on a variety of time scales and variably accessible to reflection or intentional action.

In fact, gaining expertise in freediving can be likened to a succession of stages in which practitioners’ growing proficiency exposes their bodies to more stressful environments and new adaptive challenges. Earlier progress, whether changes in technique, altered perceptions or blunted neurological responses, set up more severe environmental conditions within which further physiological adaptations arise. In other work on cultural canalization of neuroplasticity, this process has been called a ‘behavioural-developmental spiral’ because alterations occur at multiple levels, from the volitional and perceptual to the behavioural and physiological [27,28]. A changed perception, like diminished reactivity to hypercapnia, for instance, might allow a diver to extend breath holds, provoking more severe bradycardia or elevating hypoxic stress, possibly leading in the long term to enlargement of the spleen. In this model, ‘cultural embodiment’ is not a one-way process, but the complex dynamics that induce and maintain patterns of human phenotypic variation from the plasticity in us as organisms.

Rather than partition the influences on phenotypic expression between environmental and behavioural factors, I stress that they are intertwined from the start. Cultural anthropologists interested in embodiment have done a remarkable job of documenting the broad diversity of bodily practices; these cultural practices are part of the developmental niche within which distinctive forms of human variation are cultivated by different groups. Rather than seeing embodiment as the incarnation of abstract cultural symbols or ideas, this approach to embodiment emphasizes that culturally patterned life worlds, including patterns of attention, linguistic practice, repeated behaviours and activity-based niches, have cumulative phenotypic consequences. Some cultural variations can be made explicit, ideational, linguistic or cognitive. However, embodying skills leads to the biasing and canalization of organic processes of development, the alteration of sensory acuteness, the development of strategies for action or its inhibition, the subtle shifting of boundaries of proprioceptive control or the automatization of action patterns. Culture is embodied not only by working against biology but also by influencing biological patterns of development and their outcomes.

## Data Availability

This article has no additional data.
